# A Behavioral Measure of Costly Helping: Replicating and Extending the Association with Callous Unemotional Traits in Male Adolescents

**DOI:** 10.1371/journal.pone.0151678

**Published:** 2016-03-15

**Authors:** Joseph T. Sakai, Manish S. Dalwani, Susan K. Mikulich-Gilbertson, Shannon K. McWilliams, Kristen M. Raymond, Thomas J. Crowley

**Affiliations:** Division of Substance Dependence, Department of Psychiatry, University of Colorado School of Medicine, Denver, Colorado, United States of America; University of Granada, SPAIN

## Abstract

**Background:**

Some conduct-disordered youths have high levels of callous unemotional traits and meet the DSM-5’s “with limited prosocial emotions” (LPE) specifier. These youths often do aggressive, self-benefitting acts that cost others. We previously developed a task, the AlAn’s game, which asks participants to repeatedly decide whether to accept or reject offers in which they will receive money but a planned charity donation will be reduced. In our prior work, more "costly helping" (*i*.*e*., rejecting the offered money and protecting the donation) was associated with lower callous unemotional traits. Here we extend that prior work in a larger sample of adolescent male patients with serious conduct problems and controls, and test whether this association is mediated specifically by a Moral Elevation response (*i*.*e*., a positive emotional response to another’s act of virtue).

**Methods:**

The adolescent male participants were: 45 patients (23 with LPE) and 26 controls, who underwent an extensive phenotypic assessment including a measure of Moral Elevation. About 1 week later participants played the AlAn’s game.

**Results:**

All AlAn’s game outcomes demonstrated significant group effects: (1) money taken for self (p = 0.02); (2) money left in the charitable donation (p = 0.03); and, (3) costly helping (p = 0.047). Controls took the least money and did the most costly helping, while patients with LPE took the most money and did the least costly helping. Groups also significantly differed in post-stimulus Moral Elevation scores (p = 0.005). Exploratory analyses supported that the relationship between callous unemotional traits and costly helping on the AlAn’s game may be mediated in part by differences in Moral Elevation.

**Conclusions:**

The AlAn's game provides a standardized behavioral measure associated with callous unemotional traits. Adolescents with high levels of callous unemotional traits engage in fewer costly helping behaviors, and those differences may be related to blunting of positive emotional responses.

## Introduction

Conduct disorder, characterized by aggression to people and animals, rule breaking, lying and conning others [1], is common, affecting about 6–10% of U.S. adolescents [2, 3]. Youths with conduct disorder are very likely to try alcohol and illicit substances early in life [4], to develop early-onset substance use disorders [5], and to engage in HIV-risk behaviors [6]. This group has high rates of mortality [7–11], represents a major source of violent behavior [12, 13], is likely to be incarcerated [14, 15], and is very costly to society [16]. However, conduct disorder is a heterogenous phenotype. About 40% of such children will develop an adult diagnosis of antisocial personality disorder, but more than half will not [17], with some apparently remitting from their problem behaviors [18]. Given this heterogeneity, several strategies have been employed to subtype conduct-disordered youths [19], and in recent years a large body of research has focused on callous-unemotional traits. Callous unemotional traits describe individuals who show a callous lack of empathy, who lack remorse or guilt, who are unconcerned about their performance, and who exhibit shallow or deficient affect [1]. Children with conduct disorder and high callous unemotional traits display more aggressive behaviors [20], tend to be more refractory to treatment [21, 22] and have more persistent courses of their antisocial behavior problems[20]. Thus, conduct disorder plus high callous unemotional traits may represent an important subtype of conduct disorder, prompting the inclusion of a “with limited prosocial emotions” (LPE) specifier to the conduct disorder diagnosis in DSM-5 [1].

Several models have been proposed to explain high rates of aggressive and violent behavior among psychopathic individuals and children with LPE [23, 24]. One recent influential model by Blair and colleagues [25, 26] proposes that emotional differences undergird behavioral differences. Youths with LPE demonstrate a “deafness” to displays of sadness and fear [27, 28], are less reactive to pain experienced by others [29], and are less responsive to distress cues of others [30]. Therefore, when others display fear, sadness, pain and/or distress, those with LPE lack emotional cues that would guide them to restrain aggressive and antisocial behaviors. In contrast, those with conduct disorder without LPE are proposed to exhibit quick and exaggerated emotional responses to perceived perturbations (e.g., threat) that drive reactive aggressive behaviors [25]. These differences in emotional reactivity then are proposed to mediate the link between conduct disorder, LPE and aggressive antisocial behaviors.

Youths with serious conduct problems also differ in prosocial behaviors [31]. However, more limited research has been designed to examine these behavioral differences. Nonetheless, Blair's model might be extended to posit that differences in prosocial behaviors are mediated by differences in other emotional domains, such as positive emotions. One likely candidate positive emotion that might influence prosocial behaviors is Moral Elevation. According to Algoe & Haidt, [32] people may experience a positive response to another’s act “of charity, gratitude, fidelity, generosity or any other strong display of virtue”. This reaction may occur even though the observer is not involved in, and does not benefit from, these positive behaviors. Haidt [33] carefully outlined a constellation of emotional, physical and psychological aspects of a Moral Elevation response. To date it has been demonstrated that Moral Elevation can be elicited with stimulus stories or videos [32], that the Moral Elevation response can be measured with questionnaires [34, 35], and that the experience of Moral Elevation is associated with subsequent prosocial and affiliative behaviors [32]. Here for the first time, we examine whether reductions in Moral Elevation may characterize the behavior of youth with serious conduct problems and LPE, as compared to youth with serious conduct problems but without LPE, or youth without such problems.

Although many studies use self-reports to examine outcomes of interest (aggressive or prosocial behavior), laboratory-based behavioral measures may have certain advantages, given that conduct disorder is characterized by lying. Considering this, we developed and pilot tested a behavioral measure, the AlAn’s game, and demonstrated that game outcomes are associated with severity of callous unemotional traits [36]. The rationale for the task derived from prior work on costly punishment among other related concepts [37–41]. This work has shown that individuals, when asked to consider a benefit to themselves and/or a benefit to another, are not simply driven by self-interest, but instead are willing to impose punishment on “bad actors” even when there is a cost to themselves [42]. This common human tendency, called "costly punishment", has been demonstrated worldwide [43] and has been proposed as an important mechanism of social cooperation [44]. In contrast, giving is increased when directed toward an agent generally considered to be “deserving” or beneficent [41]. Drawing from the work of Moll et al., [39] we designed the AlAn’s game where the player must simultaneously consider a benefit to themselves and a cost to a charity that is generally considered beneficent. The AlAn’s game makes a series of offers in which players will receive money but a planned charitable donation will be reduced; players must accept or reject each offer. In our prior work most youths, even those with LPE, declined at least some offers, helping the charity at a cost to themselves, a behavior we term "costly helping". But why would youth engage in costly helping and not just maximize self-benefit? We told all participants that how they played the game would be held in strict confidence. Therefore concerns about reputation [45] were unlikely to drive this behavior. Likewise, participants knew this charity, the Red Cross, would never be informed how large a donation each player left while playing the game, reducing concerns that hopes for reciprocal altruism [46] drove choices in the game. When individuals must consider reward to self and cost to another simultaneously, they appear to sublimate drive for self and consider the characteristics of the other and “good actors” are helped (costly helping), at a cost to oneself. But people differ in the extent of costly helping they exhibit. Our prior work suggests that LPE is associated with lower levels of costly helping [36]. Here we seek to confirm the negative association between LPE and this prosocial behavior, costly helping.

In this study we sought to (1) replicate our prior work showing that LPE (i.e. high callous unemotional traits) reduces costly helping, (2) extend that prior work by testing the prediction that costly helping will not be reduced in individuals with serious conduct problems but without LPE, (3) test whether groups (controls, conduct problems, conduct problems with LPE) differ in Moral Elevation and (4) test whether differences in Moral Elevation mediate the link between callous unemotional traits and costly helping. Given that this study is the first (to our knowledge) to examine Moral Elevation and costly helping in youth with conduct problems and LPE, we sought to avoid a potential confound of sex differences in our design by limiting it to males only. For example, conduct disorder has a male:female ratio of about 2–4:1, but early-onset conduct disorder that persists into adulthood has a ratio of 10–15:1 [47–50]. Similarly sex differences in self-reported empathy [51], prosocial reasoning [52] and level of callous unemotional traits [53] have been demonstrated. As males predominately commit violent criminal behaviors [54, 55], our sample was limited to them.

## Materials and Methods

This study protocol was approved by the Colorado Multiple Institutional Review Board (Protocol #12–0117). Sample selection: Patients were recruited from a university-based adolescent treatment program for youth with serious externalizing behavior problems including substance and conduct problems. Controls were recruited via online advertising (e.g., Craigslist) from zip codes from which patients are usually referred.

### Sample Selection

Inclusion Criteria for all adolescent participants: (1) male; (2) estimated full-scale IQ ≥ 80; (3) 15–18 years of age; (4) free of past-week illicit drug or alcohol use evidenced by two negative urine drug and saliva alcohol tests and free of last 30 days illicit drug or alcohol use by self-report; (5) adolescent and all first-degree relatives have never worked, volunteered for or received assistance from the Red Cross; (6) valid written consent from parent, together with written assent from the participant (or written consent from participants 18 years of age); (7) patient and parent have adequate English and reading proficiency to provide informed consent or assent and to complete study measures and play the AlAn’s game. Because all participants underwent an MRI while playing the AlAn’s game and had resting state and structural MR imaging, these additional inclusion criteria were imposed: (8) right handed; (9) no physical illness that would prevent participation or that has a well-documented association with brain morphometric changes; (10) no reports (or evidence) of marked claustrophobia; (11) no orthodontic braces or other devices; (12) no other contraindications to MRI scanning, including intracranial, intraorbital, or intraspinal metal, pacemakers, cochlear implants or other non-MRI-compatible devices; (13) no history of head injury with loss of consciousness for more than 15 minutes, neurological illness, or history of neurosurgical procedures; (14) no red-green color blindness. Data on neuroimaging results are discussed in a separate report.

Patient-specific Inclusion Criteria were: (1) adolescent in treatment for serious substance and conduct problems; (2) at least one non-nicotine DSM-IV substance use disorder. *Control-specific Inclusion Criteria were*: (1) no history of court conviction for offenses other than minor traffic or curfew violations; (2) no history of substance-related treatment or substance-related expulsion from school; (3) does not meet DSM-5 conduct disorder symptom threshold (has < 3 conduct disorder symptoms lifetime); (4) does not meet criteria for non-nicotine substance use disorder; (5) does not meet the "with limited prosocial emotions" specifier for the conduct disorder diagnosis in DSM-5. Exclusion Criteria for all adolescent participants were: (1) psychotic, bipolar, or anxiety disorder, as indicated by assessment and confirmed by clinical interview (JTS); (2) obvious intoxication; (3) current risk of suicide, violence, or fire setting sufficiently great to interfere with evaluation or to endanger evaluators; (4) currently experiences caffeine/nicotine withdrawal with cessation of nicotine or caffeine use.

### Study Procedures

#### Meeting 1

All adolescent participants completed:

1. race and ethnicity questions, as requested by granting agencies;
2. screening forms to assess for MRI contraindications and history of neurological illness;
3. Vocabulary and Matrix Reasoning subtests of the Wechsler Abbreviated Scale of Intelligence (WASI[56]), from which we estimated full scale IQ;
4. NIMH Diagnostic Interview Schedule for Children-Version IV (DISC-IV [57]), a fully-structured computer-assisted interview for youths which provides DSM-IV diagnoses for co-morbid mental health disorders;
5. Conduct-Disorder Supplement, which gathers information on lifetime conduct disorder symptoms [12];
6. Composite International Diagnostic Interview-Substance Abuse Module (CIDI-SAM; [58], a structured, computerized instrument providing valid [59] diagnoses of adolescent [12] DSM-IV substance abuse or dependence;
7. CIDI-SAM Drug Supplement [12]. This assessment obtains additional information regarding drug use, and allows us to determine whether participants are at risk for caffeine and nicotine withdrawal;
8. Inventory of Callous and Unemotional Traits (ICU; [60]). Each item was scored 0–3 with higher scores indicating higher levels of callous unemotional traits (i.e. after reverse scoring appropriate items). These were summed to create a total ICU score (range 0–72; note: Items from ICU were used to determine if subjects met the DSM-5 LPE specifier [36]; see Section “Two Patient groups” below for details);
9. Peak Aggression Scale, a structured procedure [12] for rating the youth’s self-reported most aggressive lifetime behavior;
10. Youth Self Report (YSR;[61]). This standard assessment produces dimensional ratings of conduct, attention, and affective problems, with excellent reliability and validity;
11. A urine sample for on-site drug testing (AccuTest^™^; Jant Pharmacal Corp., Encino, CA) and a saliva test for alcohol (AccuStrip^™^; Jant Pharmacal corp., Encino, CA); and
12. Moral Elevation was measured utilizing the following procedures. First, participants completed a self-report pre-stimulus Moral Elevation questionnaire (modified from [34, 35]; available upon request). Then participants viewed a short video about an uncommon act of selflessness, that has been previously used as a stimulus to elicit Moral Elevation ([62]; http://www.youtube.com/watch?v=e9JcX2X7XnM), and afterwards they completed the full Moral Elevation post-test measure [29, 34, 35]. This measure of Moral Elevation was added to the battery after the start of the study, after the first 15 participants had been assessed.

Parents also completed a brief set of questionnaires regarding their child and their family, including a standard rating of socioeconomic status [12, 63] and the Child Behavior Checklist (CBCL) that produces dimensional ratings of conduct, attention, and affective problems, with excellent reliability and validity, [64].

#### Meeting 2

Approximately 7 days after the first assessment, adolescent participants were transported via taxi to the Brain Imaging Center of the University of Colorado Denver on the Anschutz Medical Campus. They then submitted a urine test for drugs and a saliva test for alcohol, and a trained research assistant administered a structured interview about any recent caffeine or nicotine consumption, alcohol and drug use, and current nicotine or caffeine withdrawal symptoms. In a quiet, private research-dedicated testing room participants viewed a 90-second investigator-produced Power Point slide presentation with pre-recorded messages and pictures showing good things the Red Cross does. Participants then indicated how much good they feel the Red Cross does by marking on a 100mm line anchored by the terms “No good at all” and “Lots of good” (Red Cross Visual Analogue Scale). Participants were then trained to play the AlAn’s game. Another automated Power Point presentation with pre-recorded audio explained the game’s instructions; participants then answered 2 questions about specific trials of the game to ensure comprehension of instructions, and played a short practice version of the game (with dollar amounts not found in the real game) on a laptop to acclimatize to the game timing and format. Participants were reminded that research data would be held in strict confidence by the researchers and that parents/treatment program staff would not be told the choices they made while playing the game. Participants were given the opportunity to ask questions and to replay the practice game as needed. Participants then were taken to our mock scanner where they could acclimatize to the MRI environment. They played the short practice version of the game while lying in the mock scanner and listened to recorded MRI machine sounds through earphones. Then participants played the AlAn’s game while in the MRI scanner (manuscripts detailing the MRI findings are in preparation). Participants were reimbursed $80 at the end of the MRI session and could earn (and keep) up to approximately 16 additional dollars while playing the AlAn’s game if they chose to accept all trials where they could benefit even if the Red Cross donation would be reduced. Parents were also reimbursed $10 for completing questionnaires about their child.

### Description of the AlAn’s Game

**Fig 1:**
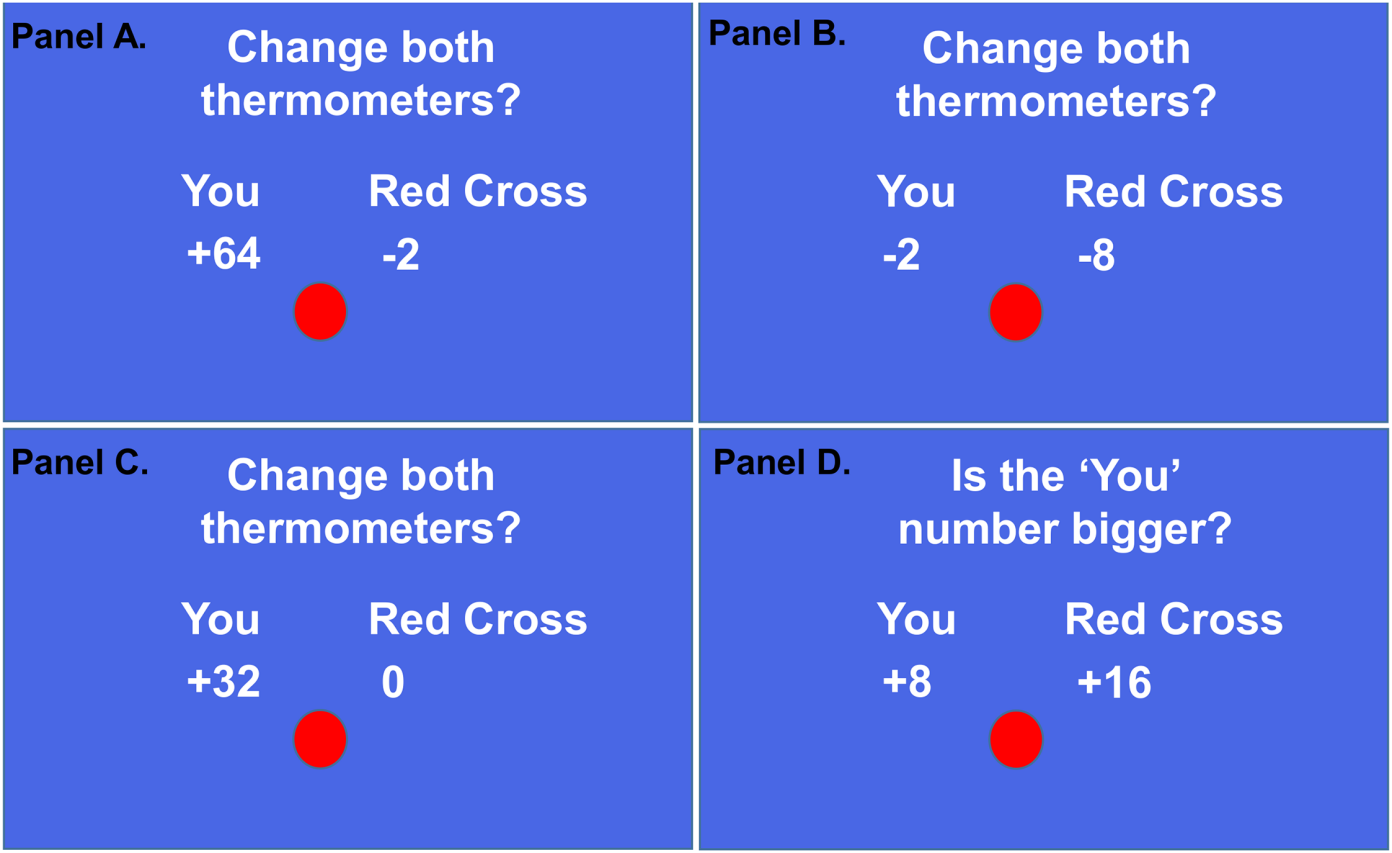
Examples of the different trial types in the AlAn’s game. Panel A shows an Active Trial where the participant will receive 64 cents and the Red Cross donation will be reduced by 2 cents. Participants are asked to accept or reject this offer. Panel B shows an Attentional Control Trial where the participant will lose 2 cents and the Red Cross donation will be reduced by 8 cents. We term this kind of Attention Control trials, “Logically-Reject” Trials. Panel C shows an Attention Control Trial where the participant will gain 32 cents and the Red Cross donation won’t change. We term these “Logically-Accept” Trials. Panel D shows a Calculation Trial where the You number (+8) is not bigger than the Red Cross number +16). Note: The circle remains red for 5 seconds, allowing participants to view the trial content. Then the circle turns green and subjects have 1 second to press either yes (accept) vs. no (reject).

The AlAn’s (**Al**triusm/**An**tisocial) game requires participants to make decisions to accept or reject real-money offers in which they will benefit, but at a cost to another [36]. A planned, real donation to the Red Cross starts at $16 but can decline due to choices made by the participant playing the game; participants start with no money. We told participants that we would give the Red Cross the value of the charity donation remaining at the end of playing the game (we donated $612.36 to the Red Cross at the end of the study). There are 144 choices (“trials”) in the AlAn’s game. During each trial participants 1) view and consider the offer, 2) press an “accept” key or a “reject” key, 3) observe the outcome of the choice. Analogue “thermometers” and digital counters show how much money the participant has accrued and the current remaining value of the charity donation. There are 3 trial types in the AlAn’s game (see Fig 1): **Active Trials** present the amount that the participant will gain (range in different trials 2–64 cents) and amount the Red Cross will lose (same range but usually different amount). Participants accept or reject each choice; after rejected choices neither counter changes. **Attentional Control Trials** have a predictable response. Participants should be motivated to reject all trials in which both they and the Red Cross will lose money (hereafter referred to as Logically-Reject Trials) and conversely, should be motivated to accept all choices in which they will get money but the Red Cross donation will not be reduced (hereafter referred to as Logically-Accept Trials). **Calculation Trials** assess whether patients and controls can understand the relative numerical values in the time allotted. They present one positive number for the participant and another for the Red Cross and the participant is asked to determine, “Is the YOU number bigger?” Participants press the YES button to indicate that the participant’s number is bigger than the Red Cross’ number. Calculation Trials use the same matrix of numbers used in the Active Trials. As an incentive, if subjects correctly answered at least 75% of the 60 Calculation Trials, they received an additional 25 cents.

### Two Patient Groups

By design, we sought to recruit adolescent patients with serious conduct problems and divide them into two groups: those meeting the LPE specifier and those not meeting this specifier. Therefore, patients recruited for the study were divided into those meeting and not meeting this specifier by utilizing questions 3, 5, 6, and 8 from the ICU, following our prior procedures [36]. Based on our prior work, we expected that while using these procedures about half of the patient sample would meet the LPE and that these two groups would be very similar for substance use disorder severity. It is important to note that we utilize the LPE categorization method from the ICU described in the supporting document of the DSM-5 revision [65], and this is the same procedure we used in our prior work on the AlAn’s game [36], but others in the research literature have used other symptoms sets or other thresholds for item endorsement [66, 67].

### Main Outcome Measures

For the AlAn’s game, we had 3 *a priori* outcome measures of interest: (1) the amount of money taken for self while playing the game, (2) the amount of money remaining in the Red Cross donation after playing the game and (3) the number of times participants did not take the Active Trials. The game automatically produces the number of accepted Active + Attention Control Trials. Therefore, this third variable was calculated by the following formula (= 72-(number of total trials accepted—the number of accepted Attention Control Trials). This final outcome served as our measure of “costly helping.”

### Analytic Plan

We compared patients with LPE, patients without LPE and controls for demographics (age, race, ethnicity, SES), estimated IQ, clinical and diagnostic measures (substance use disorders, conduct disorder severity, peak aggression) and callous unemotional traits severity, utilizing chi square and Fisher Exact Tests for categorical and ANOVA or Kruskal Wallis tests for dimensional variables. Next we compared groups with ANOVA or Kruskal Wallis on our 3 AlAn’s game outcome measures, and the Red Cross VAS (i.e. “How much good does the Red Cross do?”); if the group effect was significant, we evaluated all 2-way group comparisons (e.g., using Tukey’s post hoc tests). We also calculated the percent of Logically-Accept Trials accepted, percent of Logically-Reject Trials rejected, and the percent of Calculation Trials correctly answered. We then completed Spearman rank order correlations between our 3 AlAn’s game outcomes, ICU total score, conduct disorder symptom count and parent-reported CBCL and adolescent-reported YSR conduct problems scale raw scores and the attention-deficit hyperactivity problems raw scores 1) across all groups (n = 71), 2) across patient groups (n = 45) and 3) within controls (n = 26). Finally, we calculated the percent of Active Trials accepted by trial type for each groups.

To address our hypotheses that Moral Elevation would differ between the groups, we first calculated pre-stimulus (*i*.*e*., prior to viewing the video), and separately post-stimulus, scores for the four Moral Elevation domains: desire to be a better person, view of humanity, elevating emotions, and physical symptoms (each with a range of 0–4, questions available upon request from the corresponding author) and summed them to create a total Moral Elevation score (range 0–16) following the general procedures outlined by others [34, 35]. We tested for group differences in pre-stimulus total Moral Elevation using ANOVA (and post hoc Tukey comparisons, when group effect was significant). This procedure allowed us to evaluate whether groups differed significantly in Moral Elevation scores at baseline. Our design was such that had groups differed in Moral Elevation scores at baseline, we planned to use change from pre-stimulus to post-stimulus to estimate Moral Elevation response to the stimulus, adjusting for baseline differences. Because groups did not differ in Moral Elevation scores at baseline, we used post-stimulus Moral Elevation scores as our outcome and tested whether groups differed with ANOVA and post-hoc 2-group comparisons. Next, we tested within group, whether Moral Elevation scores significantly increased from pre-stimulus to post-stimulus, using paired t-tests. Finally, we conducted exploratory mediational analyses. We utilized the Preacher & Hayes [68] approach to mediational analyses using the Indirect Macro (http://www.afhayes.com/index.html) with total ICU score as the independent variable, costly helping on the AlAn’s game as the dependent variable and post-stimulus total Moral Elevation as the mediator, covarying group (patient vs. control), completing 5,000 bootstrap iterations and calculating the 95% confidence interval.

## Results

### Sample Description

Table 1 presents demographics, IQ measures and diagnostic or clinical measures for patients with LPE (n = 23), patients without LPE (n = 22) and controls (n = 26). Patients without LPE were slightly older (17.2 years) than patients with LPE and controls (both 16.5 years). Groups did not differ significantly in terms of race (p = 0.15), ethnicity (p = 0.18) or parent socioeconomic status (p = 0.10). Groups differed significantly in IQ (p = 0.047) with controls (mean estimated IQ 106.5) having the highest scores (patients with LPE 100.3 and patients without LPE 100.9) but all three post-hoc two-group comparisons were non-significant. As expected from our sampling strategy, no controls met criteria for conduct disorder or any substance use disorder but the two patient groups had similar rates of self-reported conduct and substance use disorder diagnoses. Thus, as expected from prior work (Sakai et al., in review), the two patient groups were quite similar overall for co-morbidity at the diagnostic level. However, patients with LPE averaged 6.6 lifetime conduct disorder symptoms, while patients without LPE averaged 5.0 and controls 0.3 (p<0.001). Patients with LPE also had the highest peak aggression scores (6.3; p<0.001) and ICU total scores (p<0.001), followed by patients without LPE and controls.

**Table 1:** Sample Description: Demographic, IQ and Diagnostic/Clinical Information.

	Pts-LPE (n = 23)	Pts-NoLPE (n = 22)	Cts (n = 26)	3-group test	post-hoc 2-group comparisons^a^
**Demographics**					
**Age (years)**	16.5 (0.77)	17.2 (0.84)	16.5 (0.82)	F(2,68) = 5.78; p = 0.005;	1;3
**Race**					
White (vs. other, mixed race)	15 (65.2%)	17 (77.3%)	23 (88.5%)	χ^2^(2) = 3.78; p = 0.15	
**Hispanic Ethnicity (yes)**	11 (47.8%)	7 (31.8%)	6 (23.1%)	χ^2^(2) = 3.40p = 0.18	
**Parent SES**	49.7^b^ (15.4)	45.0^b^ (14.73)	40.3 (13.93)	F(2,65) = 2.38; p = 0.10	
**Cognitive Measures**					
**WASI-Estimated-IQ**	100.3 (8.00)	100.9 (10.12)	106.5 (10.24)	F(2,68) = 3.19; p = 0.047	
**Diagnostic/Clinical Measures**					
**Meeting DSM-IV criteria for a substance abuse or dependence diagnosis**					
Cannabis	23 (100%)	21 (95.5%)	0 (0%)	χ^2^(2) = 67.0; p<0.001	1;2
Tobacco (dependence only)	16 (69.6%)	12 (54.5%)	0 (0%)	χ^2^(2) = 27.8; <0.001	1;2
Alcohol	18 (78.3%)	14 (63.6%)	0 (0%)	χ^2^(2) = 34.6; p<0.001	1;2
Cocaine	7 (31.8%)	7 (30.4%)	0 (0%)	χ^2^(2) = 10.1; p = 0.006	1(FE); 2(FE)
Total # DSM-IV Substance abuse or dependence Diagnoses	4.3 (2.07)	3.8 (2.20)	0 (0)		1;2^c^^,^^d^
**Disruptive Behavior Disorders, Aggression and ADHD**					
Whole Life Conduct Disorder Diagnosis	23 (100%)	20 (90.9%)	0 (0%)	χ^2^(2) = 63.4; p<0.001	1;2
Whole Life Conduct Disorder Symptom Count (possible range 0–15)	6.6 (2.54)	5.0 (2.40)	0.3 (0.56)	Welch F statistic (2, 31.07) = 100.13; p<0.001	1;2^d^
Peak Aggression (possible range 0–9)	6.3 (2.83)	3.9 (3.16)^e^	0.4 (1.24)	KW; p<0.001	1; 2; 3
ADHD_CBCL_	78.2 (19.76)	82.4 (17.06)	55.8^f^ (9.51)	KW; p<0.001	1; 2
ADHD_YSR_	70.2 (18.91)	77.9 (18.43)	56.4 (11.60)	KW; p<0.001	1; 2
**Callousness and Empathy Measures**					
**ICU**					
Total Score (possible range 0–72)	31.30 (6.11)	21.18 (5.36)	17.89 (6.59)	F(2,68) = 31.62; p<0.001	2;3

Mean (sd) or count (%); **Abbreviations:** ADHD_CBCL_ = Diagnostic and Statistical Manual-oriented attention-deficit hyperactivity problems raw score from the Child Behavior Checklist; ADHD_YSR_ = Diagnostic and Statistical Manual-oriented attention-deficit hyperactivity problems raw score from the Youth Self Report; Cts = controls; ICU = Inventory of Callous and Unemotional Traits; KW = Kruskal Wallis Test; LPE = utilizing questions 3,5,6 and 8 from the ICU we determined whether participants qualified for the “with limited prosocial emotions” Specifier for Conduct Disorder; NoLPE = not meeting the with limited prosocial emotions specifier; Pts = patient.

**Footnotes:**

^a^ = Post hoc 2 group comparisons were either completed with Tukey HSD (for approximately normally distributed variables), Mann-Whitney U tests (when variables were not approximately normally distributed in this sample) or the Games-Howell post-hoc test (when equality of variances could not be assumed). Note that 1 = Controls vs. patients without LPE significant (p<0.05); 2 = Controls vs. patients with LPE significant; 3 = Patients with LPE vs. patients without LPE significant.

^b^ = not all parents completed this measure (patients with LPE n = 21; patients without LPE n = 21).

^c^ = Equality of variance could not be assumed but Welch F statistic could not be calculated as control variance was equal to zero;

^d^ = Games-Howell post-hoc two group analyses where equality of variance is not assumed;

^e^ = One patient without LPE declined to complete this questionnaire (n = 21 for this cell);

^f^ = Not all parents completed the CBCL (n = 66 across groups, 41 patients, 25 controls).

### Testing for Group Differences in AlAn’s Game Outcome Measures

**Fig 2:**
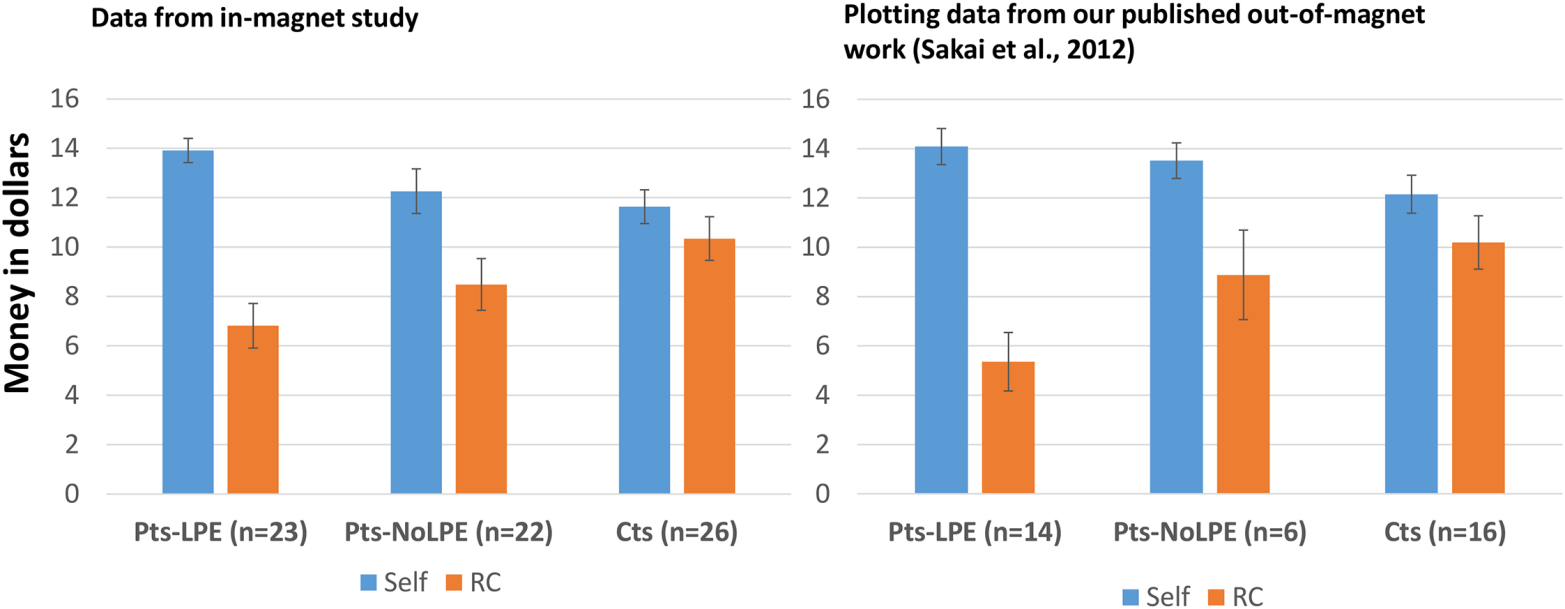
Results from the AlAn’s Game in-magnet study (left panel) and results from the previously published out-of-magnet study (right panel). Error bars indicate standard error. Money taken for self in blue and money left in the Red Cross donation in orange.

Patients with LPE, patients without LPE and controls differed significantly in the amount of money taken for self while playing the game ($13.91, $12.26, $11.64, respectively; Kruskal Wallis test p = 0.02), the amount of money left in the Red Cross donation at the end of the game ($6.81, $8.49, $10.34, respectively; F = 3.66, p = 0.03) and number of Active Trials not taken, our measure of costly helping (19.5, 28.5, 32.4, respectively; F = 3.20, p = 0.047). Because all 3-group analyses were significant, post-hoc 2-group comparisons were completed and showed that controls vs. patients with LPE differed significantly for all 3 AlAn’s outcome measures; other 2-group comparisons were non-significant at the post-hoc corrected threshold. These results are presented in S1 Table. Fig 2, left panel, shows bar graphs for the three groups (identified on the x-axis) and two AlAn’s Outcomes; Fig 2, right panel, shows the same information from our previously published work on the AlAn’s game for easy comparison (Sakai et al., 2012).

### AlAn’s Game Performance Measures

Patients with LPE, patients without LPE and controls had similar results on the Red Cross VAS “How much good does the Red Cross do?” (86.5, 91.8, 87.6, respectively, where 0 = “No good at all” and 100 = “Lots of good”; p = 0.20), suggesting that on average they viewed the Red Cross quite positively. Patients with LPE, patients without LPE and controls also accepted a high percentage of Logically-Accept Trials (95.7%, 93.9% and 97.4%, respectively; p = 0.64), rejected a high rate of Logically-Reject Trials (92.8%, 93.9% and 95.5%, respectively; p = 0.27), and correctly answered a high percentage of the 60 Calculation Trials (95%, 89.5%, 94.7% and respectively; p = 0.96).

### Additional AlAn’s Game Analyses

AlAn’s game outcomes were significantly correlated with ICU total score, lifetime conduct disorder symptom count, and parent-reported (CBCL) and adolescent-reported (YSR) conduct problems across groups; within-group correlations are also presented (see S2 Table). We also calculated and tabulated the percentage accepted for each Active Trial type within group (see S1A–S1C Fig). These additional analyses are presented for easy comparison with our prior published work (see Table 3 and S1 Figure in Sakai et al., [36] for comparisons).

### Moral Elevation

**Fig 3:**
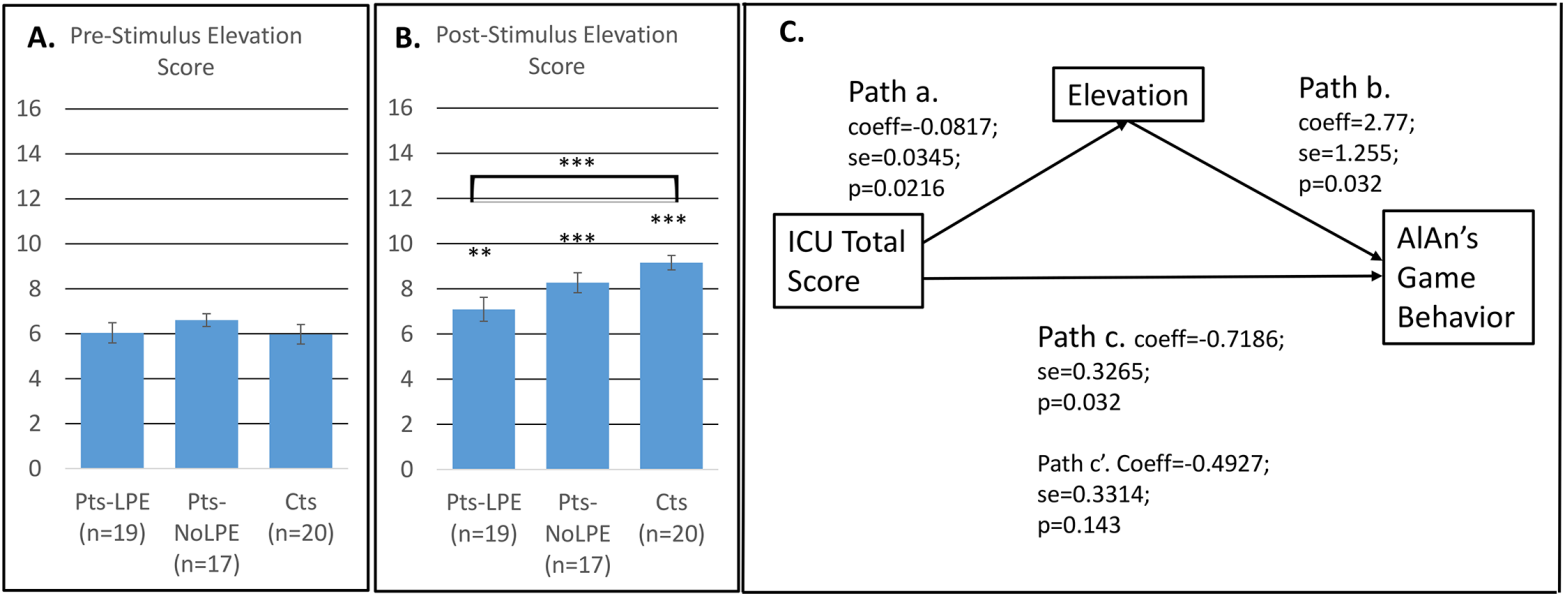
Aggregate Elevation Scores Prior to (Panel A) and Post-stimulus Administration (Panel B); error bars indicate standard errors. Mediation Model, testing whether Elevation mediates the association between level of Callous Unemotional Traits and AlAn’s Game behavior (Panel C). Bracket above bars indicates ANOVA was significant for group differences; * p<05; ** p≤0.01; *** p≤0.005. Asterisks directly above post-stimulus bars indicate that pre to post change was significant within group Panel C: Mediation model using Preacher & Hayes (2008) method with ICU total score as the independent variable, Costly helping on the AlAn’s Game as the dependent variable, Elevation post-stimulus score as the proposed mediator and controlling for patient vs. control; number of bootstrap resamples equals 5,000. 95% CI = -0.53 to -0.06 and is significant.

Groups did not differ on pre-stimulus total Moral Elevation score (Fig 3, panel A). All groups showed a significant pre- to post-stimulus increase in Moral Elevation scores (Fig 3 panel B, asterisks directly above each bar), and groups differed significantly in post-stimulus Moral Elevation scores (bracket above the bar graphs, panel B; patients with LPE mean = 7.1 (standard deviation = 2.32); patients without LPE 8.3 (1.81); controls 9.2 (1.43); F(2,53) = 5.89; p = 0.005; in 2-way comparisons patients with LPE differed significantly from controls p = 0.003; other comparisons were non-significant at post-hoc threshold). Fig 3, panel C, shows the results for analyses testing whether post-stimulus Moral Elevation total score mediated the association between total ICU score and AlAn’s game behavior (costly helping). Path “c” shows a significant relationship between total score on the Inventory of Callous Unemotional traits and costly helping. Path “a” shows a significant relationship between callous unemotional traits and Moral Elevation and path “b” shows that Moral Elevation has a unique effect on costly helping. Path “c’” shows that the association between callous unemotional traits and costly helping becomes non-significant once Moral Elevation is introduced into the model. After bootstrapping (5000 iterations), the 95% confidence interval testing for mediation effects of Moral Elevation excludes zero (95% CI -0.53 to -0.06), supporting a significant mediational effect (Preacher & Hayes, 2008). Mediational analyses within patients only (n = 36) show that the 95% CI for mediational effects of Moral Elevation remains significant (-0.86 to -0.06).

## Discussion

### Replicating and Extending Prior Work on the Link between AlAn’s Game and Callous Unemotional Traits

We replicated our prior work showing that a game of costly helping significantly discriminates patients with LPE from controls. Our findings are remarkably similar to our preliminary work (see two panels in Fig 2), suggesting a robust association while utilizing the same procedures. Examination of S1 Fig supports that the game operated as expected. Moving from left to right, we see that, while holding gain-to-self constant and increasing loss to the Red Cross, acceptance rates generally decline. Similarly, as we move from the top of each column to the bottom, we see that as the loss to the Red Cross is held constant and gain-to-self increases, the acceptance rates generally increase. Thus although the amount of money in individual trials is small (range 2–64 cents), participants’ behavior supports the idea that the amounts were motivating. In addition, similar performance between groups on Calculations trials supports the idea that groups could assess the relative values presented in the Active Trials in the time allotted. The high acceptance rates of Logically-Accept Trials and the high rates of rejection of the Logically-Reject Trials support that all groups behaved logically when unambiguous trials were presented. It also supports that subjects generally assessed trial content, instead of simply falling into a pattern of always accepting, or rejecting trials.

Just as in our preliminary work, we controlled for several confounds that might have influenced our results. Participants were monitored for substance use and were 30 days substance free per self-report and at least 1-week substance free by urine/saliva monitoring. Thus, recent substance use did not drive decision-making during the game. The game is also designed to provide concrete offers, rather than assessment of probabilities. Therefore, risk-assessment or risk-taking are eliminated from the game, which is important given patients with conduct disorder/substance use disorder have increased risk-taking propensity [69, 70]. Because patients also tend to act impulsively, the game requires that participants wait 5 seconds prior to making a choice. This delay was instituted to reduce impulsive decision making; such delays have been shown to normalize response reversal learning in adults with ASPD [71]. In addition, our correlational analyses (S2 Table) show that ADHD scores (parent- and child-reported) were not significantly related to AlAn’s game outcomes, suggesting that inattention and impulsivity did not drive behavior in the game.

This study also extends our prior work in one notable and important way. In our prior work we had relatively few patients without LPE and therefore did not include results on that group in our published work [36] but here we collected data on 22 patients without LPE. We predicted that patients without LPE would behave similarly to controls on the AlAn’s game, but instead demonstrate that the behavior of patients without LPE is intermediate between patients with LPE and controls on our three AlAn’s game outcomes of interest. This pattern was true even though patients without LPE had dimensional scores for callous unemotional traits similar to controls (see bottom row of Table 1). This finding suggests that although behavior on the AlAn’s game is associated with levels of callous unemotional traits, conduct disorder itself may convey some additional effect that reduces the propensity to engage in costly helping. For example, conduct disorder *severity* is associated with behavior on the game (see S2 Table). Future work is needed to explore this possibility.

Clinical implications: The AlAn’s game provides a standardized behavioral measure reflecting “limited prosocial emotions”. This simple game may be useful in multiple research settings where confidentiality is assured, including studies that attempt to search for biological markers of callous-unemotional traits, such as MRI studies. Unfortunately, we do not anticipate that AlAn’s game would be useful in clinical settings where patients had incentive to dissemble (e.g., court-ordered evaluations). We suspect that with incentive, players could easily alter behavior on the game.

### Explaining the Link Between Callous Unemotional Traits and AlAn’s Game Behavior

One important question is, “Why do groups differ in their behavior on the AlAn’s game?” One possibility is that groups differ in their moral judgments [72]. Unfortunately, we did not measure moral judgments or reasoning here but do show that all three groups viewed the Red Cross equally favorably, consistent with our past work [36]. Future studies might measure whether participants think the Red Cross “ought” to be helped and explicitly measure moral judgments and reasoning to test for associations with costly helping. Although there are very important exceptions [30, 72], prior work supports that adults with psychopathy may make similar moral judgments to controls [73, 74] while still differing in their behaviors [75]. If true, one logical question emerges: why would individuals with psychopathy, despite having similar moral judgments or beliefs, exhibit behaviors that do not align with those beliefs? In an attempt to answer this question, we turned to the influential model proposed by Blair et al. [26] which suggests that individuals with LPE lack an ability to interpret emotional cues, which are important to restraining aggressive and antisocial behavior. Here we sought to extend that model to help explain the linkage between callous unemotional traits and costly helping as being mediated by Moral Elevation.

We present several novel findings regarding Moral Elevation. For example, we demonstrate that a Moral Elevation response is a fairly universal phenomenon. Patients in treatment for serious antisocial behavior and substance problems who also have high levels of callous-unemotional traits (and meet the LPE specifier) do exhibit a significant Moral Elevation response (pre-stimulus to post-stimulus increase in Moral Elevation scores). But there are group differences in the magnitude of that response. Controls show the strongest Moral Elevation, patients without LPE show the next highest and patients with LPE show the weakest Moral Elevation response. Next, using the Preacher & Hayes [68] method, we demonstrated that Moral Elevation mediates the link between callous-unemotional traits and costly helping in this sample. Taken together these results support that patients with LPE may experience a blunted response to emotional cues, which means they are lacking an immediate motivator toward prosocial behavior. Individuals with a blunted Moral Elevation response may require greater vigilance and top-down cognitive control to ensure their actions match their moral beliefs. Although from Blair’s model [26] we might expect that patients without LPE would function similarly to controls or inhibit prosocial behaviors mainly in the setting of exaggerated self-focused emotionality (e.g., anxiety and personal distress), patients without LPE fell between controls and patients with LPE in their Moral Elevation response and costly helping behaviors.

### Implications for Conservation of Human Altruism

Finally, although our main focus has been on patients with LPE, our results may have a broader set of implications. Most participants while playing AlAn’s game made at least some choices to not take offers where they would have received money but at a cost to the charity donation. Why not just maximize gains for self? Here, we propose that when an actor is seen as beneficent, as a “good” actor, individuals engage in costly helping, where they give up a benefit to self to aid a beneficent other. Our data suggest most youths, even many patients with LPE, engage in some level of costly helping, which is a subset of altruistic behaviors. All costly helping is altruism but not all altruistic acts are costly helping. For example, altruistic acts may be directed randomly or based on need of the recipient, not based on whether the recipient is a “good” actor. Instead costly helping, by the definition proposed here, is directed at a good actor. Costly helping then is directed at altruists and based on our initial studies, most individuals engage in at least some costly helping. If it could be shown that costly helping, similar to costly punishment, is a human attribute common across human societies, this could have implications for the promotion of cooperation within social groups and the evolutionary conservation of human altruism.

### Limitations

Our work has several important limitations. First, we selected a male-only sample. Thus, whether our results can be extrapolated to females is an open question. However, the traits of interest (e.g., conduct problems, callous-unemotional traits) are more prevalent in males, prompting our male focus in this initial study. Second, we focus on adolescents. It is unclear whether the AlAn’s game would function similarly to assess costly helping among younger children or adults. Third, our mediational analyses should be viewed as preliminary. Ideally others might utilize general population sampling strategies rather than our selected patient-control group approach. In addition, the term mediation implies a causal relationship, where variables may be measured across time in the order proposed by the hypothesized model. Our study was not longitudinal, though we measured Moral Elevation about 1 week before playing the AlAn’s game and our independent variable, callous unemotional traits, is generally considered a stable trait [76]. Ideally the independent variable also might be manipulated (e.g., treatment vs. no treatment) allowing control of unmeasured confounders through randomization. Thus, we cannot be certain about a causal relationship in our mediational analyses. Still, despite these limitations, we hope that our work will motivate others to explore this interesting observation. Fourth, as noted above, we did not measure moral beliefs and judgments, making it impossible to rule out that differences in moral decision making explained behavioral differences in patients with LPE. Fifth, there are certainly other domains of interest which may mediate the link between callous unemotional trait and costly helping. For example, reward dominance [77] has been previously linked with antisocial behavior problems and could be tested in social (e.g., reputation) and non-social contexts. We did not measure these constructs here. Future research might explore such links. Sixth, we did not debrief subjects after they played the game to allow them to explain their behaviors. Therefore, we cannot comment on the participants’ reasoning behind various behavioral patterns. Seventh, our sample is relatively modest in size and our results therefore should be replicated in larger samples.

## Conclusions

We recruited three groups of male adolescents: patients with serious conduct and substance problems and LPE, patients with serious antisocial behavior problems but without LPE, and controls. We replicated our prior finding that the AlAn’s game significantly discriminates patients with LPE and controls in their behavior, showed that self-reported post-stimulus Moral Elevation strongly discriminates groups and in our exploratory analyses demonstrated that Moral Elevation mediated the link between callous-unemotional traits and costly helping behaviors in this sample. However, our sample size is relatively modest in size and our results require independent replication. Therefore, we now make the AlAn’s game E-Prime^®^ (Psychology Software Tools, Sharpsburg, PA) program available to other laboratories with the hopes that new groups may attempt to replicate this association between callous unemotional traits and costly helping among adolescents (game may be obtained by contacting authors Sakai or Dalwani).

## Supporting Information

S1 Fig Acceptance rates for each Active Trial: Patients with LPE, Patients without LPE and Controls. Matrix of Active Trials: Percent of “Yes” or Accepted Offers by Trial Type—Active Trials displayed by trial type. For example, upper-right cell of Table A indicates that 41% of the time patients with LPE accepted trials where they could get 2 cents and the Red Cross donation would go down by 64 cents (see Supplemental Fig 1 in Sakai et al., 2012 for between-study comparisons).
(DOCX)

S1 Table Between Group Comparisons: AlAn’s Game and Related Measures. Mean (sd); Abbreviations: Cts = controls; KW = Kruskal Wallis Test; LPE = utilizing questions 3,5,6 and 8 from the ICU we determined whether participants would qualify for the “with limited prosocial emotions” Specifier for Conduct Disorder; NoLPE = not meeting the with limited prosocial emotions specifier; Pts = patient; VAS = Visual Analogue Scale. Footnotes: ^a^ Participants are asked “How much good does the Red Cross do?”, scale is measured from 0–100 with 0 = “No good at all” and 100 = “Lots of good”; ^b^ = Post hoc 2 group comparisons were either completed with Tukey HSD (for approximately normally distributed variables) or Mann-Whitney U tests. Note that 1 = Controls vs. patients without LPE significant (p<0.05); 2 = Controls vs. patients with LPE significant; 3 = Patients with LPE vs. patients without LPE significant.
(DOCX)

S2 Table Spearman Rank-Order Correlations of AlAn’s Game Outcomes with Callous Unemotional Traits, and Conduct Disorder Symptoms (see Table 3 from Sakai et al., 2012 for between-study comparisons). *p<0.05; ** p<0.01. ^a^ = Not all parents completed the CBCL (n = 66 across groups, 41 patients, 25 controls). Abbreviations: ADHDCBCL = Diagnostic and Statistical Manual-oriented attention-deficit hyperactivity problems raw score from the Child Behavior Checklist; ADHDYSR = Diagnostic and Statistical Manual-oriented attention-deficit hyperactivity problems raw score from the Youth Self Report; CDSx = Whole life conduct disorder symptom count; Costly Helping = the number of Active Trials not taken by participants (range 0–72); CP_CBCL_ = Diagnostic and Statistical Manual-oriented conduct problems scale raw score from the Child Behavior Checklist; CP_YSR_ = Diagnostic and Statistical Manual-oriented conduct problems scale raw score from the Youth Self Report; ICU_Total_ = total score from the Inventory of Callous and Unemotional Traits.
(DOCX)

S3 Table The Order of Presentation of All Trials in the AlAn’s Game. First column shows the “You” number. Second column shows the “Red Cross” number. Third column indicates trial type. ACT = Active Trials, where you will get some money (in cents) but the Red Cross donation will go down (indicate by negative number in the second column). INACT = Calculation Trials where you must determine “Is the You Number Bigger?”. ATT.YES = Logically-Accept Trials, where the subject will get money but the Red Cross donation will not be reduced. ATT.NO = Logically-Reject Trials, where both the subject and the Red Cross will lose money.
(XLSX)
