# Supplementary material for: A Behavioral Measure of Costly Helping: Replicating and Extending the Association with Callous Unemotional Traits in Male Adolescents
Source: PLoS One. 2016 Mar 15;11(3):e0151678. doi: 10.1371/journal.pone.0151678 (PMC4792436; doi:10.1371/journal.pone.0151678)
Supplement: S1 Fig — Matrix of Active Trials: Percent of “Yes” or Accepted Offers by Trial Type—Active Trials displayed by trial type. For example, upper-right cell of Table A indicates that 41% of the time patients with LPE accepted trials where they could get 2 cents and the Red Cross donation would go down by 64 cents (see Supplemental Fig 1 in Sakai et al., 2012 for between-study comparisons). (DOCX) [file pone.0151678.s001.docx]

**Supplemental Figure 1.** Acceptance rates for each Active Trial: Patients with LPE, Patients without LPE and Controls. Matrix of Active Trials: Percent of “Yes” or Accepted Offers by Trial Type – Active Trials displayed by trial type. For example, upper-right cell of Table A indicates that 41% of the time patients with LPE accepted trials where they could get 2 cents and the Red Cross donation would go down by 64 cents (see Supplemental Fig 1 in Sakai et al., 2012 for between-study comparisons).

| +2 | 78% | 65% | 50% | 39% | 30% | 41% |
| --- | --- | --- | --- | --- | --- | --- |
| +4 | 91% | 78% | 54% | 52% | 39% | 41% |
| +8 | 93% | 87% | 80% | 54% | 41% | 39% |
| +16 | 96% | 91% | 91% | 80% | 61% | 61% |
| +32 | 96% | 96% | 96% | 93% | 83% | 61% |
| +64 | 100% | 100% | 91% | 91% | 93% | 87% |
|  | -2 | -4 | -8 | -16 | -32 | -64 |

1. Patients With LPE (n=23)

You Get

Red Cross Loses (cents)

1. Patients Without LPE (n=22)

| +2 | 52% | 52% | 36% | 27% | 23% | 30% |
| --- | --- | --- | --- | --- | --- | --- |
| +4 | 66% | 52% | 30% | 45% | 30% | 23% |
| +8 | 82% | 64% | 59% | 41% | 34% | 27% |
| +16 | 75% | 73% | 70% | 68% | 41% | 50% |
| +32 | 84% | 82% | 86% | 64% | 68% | 45% |
| +64 | 80% | 82% | 82% | 84% | 75% | 77% |
|  | -2 | -4 | -8 | -16 | -32 | -64 |

You Get

Red Cross Loses (cents)

1. Controls (n=26)

| +2 | 58% | 38% | 33% | 17% | 15% | 21% |
| --- | --- | --- | --- | --- | --- | --- |
| +4 | 71% | 63% | 27% | 35% | 17% | 13% |
| +8 | 85% | 75% | 58% | 31% | 25% | 17% |
| +16 | 88% | 85% | 81% | 63% | 27% | 29% |
| +32 | 90% | 87% | 88% | 69% | 52% | 33% |
| +64 | 96% | 92% | 81% | 87% | 75% | 56% |
|  | -2 | -4 | -8 | -16 | -32 | -64 |

You Get

Red Cross Loses (cents)
