# Supplementary material for: A Behavioral Measure of Costly Helping: Replicating and Extending the Association with Callous Unemotional Traits in Male Adolescents
Source: PLoS One. 2016 Mar 15;11(3):e0151678. doi: 10.1371/journal.pone.0151678 (PMC4792436; doi:10.1371/journal.pone.0151678)
Supplement: S1 Table — Mean (sd); Abbreviations: Cts = controls; KW = Kruskal Wallis Test; LPE = utilizing questions 3,5,6 and 8 from the ICU we determined whether participants would qualify for the “with limited prosocial emotions” Specifier for Conduct Disorder; NoLPE = not meeting the with limited prosocial emotions specifier; Pts = patient; VAS = Visual Analogue Scale. Footnotes: a Participants are asked “How much good does the Red Cross do?”, scale is measured from 0–100 with 0 = “No good at all” and 100 = “Lots of good”; b = Post hoc 2 group comparisons were either completed with Tukey HSD (for approximately normally distributed variables) or Mann-Whitney U tests. Note that 1 = Controls vs. patients without LPE significant (p<0.05); 2 = Controls vs. patients with LPE significant; 3 = Patients with LPE vs. patients without LPE significant. (DOCX) [file pone.0151678.s002.docx]

**Supplemental Table 1.** Between Group Comparisons: AlAn’s Game and Related Measures

|  |  | **Pts-LPE** (n=23) | **Pts-NoLPE** (n=22) | **Cts** (n=26) | **3-group test** | **post-hoc 2-group comparisons^b^** |
| --- | --- | --- | --- | --- | --- | --- |
| **AlAn’s Game Outcomes:** | Participants’ mean earnings | 13.91 (2.33) | 12.26 (4.23) | 11.64 (3.48) | KW; p=0.02 | 2 |
|  | Mean final Red Cross donation | 6.81 (4.32) | 8.49 (4.89) | 10.34 (4.49) | F(2,68)=3.66; p=0.03 | 2 |
|  | Costly Helping (# Active Trials not taken) | 19.5 (15.07) | 28.5 (20.86) | 32.4 (18.04) | F(2,68)=3.20; p=0.047 | 2 |
| **“How Much Good Does the Red Cross Do?”** | Red Cross VAS ^a^ | 86.5 (10.36) | 91.8 (8.94) | 87.6 (11.12) | F(2,68)=1.67; p=0.20 |  |

Mean (sd); **Abbreviations:** Cts=controls; KW = Kruskal Wallis Test; LPE = utilizing questions 3,5,6 and 8 from the ICU we determined whether participants would qualify for the “with limited prosocial emotions” Specifier for Conduct Disorder; NoLPE = not meeting the with limited prosocial emotions specifier; Pts = patient; VAS = Visual Analogue Scale.

**Footnotes:** ^a^ Participants are asked “How much good does the Red Cross do?”, scale is measured from 0-100 with 0 = “No good at all” and 100 = “Lots of good”; ^b^ = Post hoc 2 group comparisons were either completed with Tukey HSD (for approximately normally distributed variables) or Mann-Whitney U tests. Note that 1=Controls vs. patients without LPE significant (p<0.05); 2=Controls vs. patients with LPE significant; 3=Patients with LPE vs. patients without LPE significant.
