# Supplementary material for: A Behavioral Measure of Costly Helping: Replicating and Extending the Association with Callous Unemotional Traits in Male Adolescents
Source: PLoS One. 2016 Mar 15;11(3):e0151678. doi: 10.1371/journal.pone.0151678 (PMC4792436; doi:10.1371/journal.pone.0151678)
Supplement: S2 Table — *p<0.05; ** p<0.01. a = Not all parents completed the CBCL (n = 66 across groups, 41 patients, 25 controls). Abbreviations: ADHDCBCL = Diagnostic and Statistical Manual-oriented attention-deficit hyperactivity problems raw score from the Child Behavior Checklist; ADHDYSR = Diagnostic and Statistical Manual-oriented attention-deficit hyperactivity problems raw score from the Youth Self Report; CDSx = Whole life conduct disorder symptom count; Costly Helping = the number of Active Trials not taken by participants (range 0–72); CPCBCL = Diagnostic and Statistical Manual-oriented conduct problems scale raw score from the Child Behavior Checklist; CPYSR = Diagnostic and Statistical Manual-oriented conduct problems scale raw score from the Youth Self Report; ICUTotal = total score from the Inventory of Callous and Unemotional Traits. (DOCX) [file pone.0151678.s003.docx]

**Supplemental Table 2.** Spearman Rank-Order Correlations of AlAn’s Game Outcomes with Callous Unemotional Traits, and Conduct Disorder Symptoms (see Table 3 from Sakai et al., 2012 for between-study comparisons)

| **BOTH GROUPS (n=71)** | | | | | | | | | |
| --- | --- | --- | --- | --- | --- | --- | --- | --- | --- |
|  | **Participants’ mean earnings** | **Mean final Red Cross donation** | **Costly Helping** | **ICU_Total_** | **CDSx** | **CP_CBCL_^a^** | **CP_YSR_** | **ADHD_CBCL_^a^** | **ADHD_YSR_** |
| Participants’ mean earnings | 1 | -0.89** | -0.92** | 0.35** | 0.31** | 0.25* | 0.27* | 0.08 | 0.09 |
| Mean final Red Cross donation |  | 1 | 0.98** | -0.37** | -0.35** | -0.29* | -0.28* | -0.10 | -0.16 |
| Costly Helping |  |  | 1 | -0.35** | -0.31** | -0.27* | -0.23 | -0.07 | -0.08 |
| ICU_Total_ |  |  |  | 1 | 0.49** | 0.35** | 0.45** | 0.30* | 0.38** |
| CDSx |  |  |  |  | 1 | 0.59** | 0.73** | 0.43** | 0.45** |
| CP_CBCL_ |  |  |  |  |  | 1 | 0.58** | 0.79** | 0.41** |
| CP_YSR_ |  |  |  |  |  |  | 1 | 0.51** | 0.65** |
| ADHD_CBCL_ |  |  |  |  |  |  |  | 1 | 0.49** |
| **WITHIN PATIENTS (n=45)** | | | | | | | | | |
|  | **Participants’ mean earnings** | **Mean final Red Cross donation** | **Costly Helping** | **ICU_Total_** | **CDSx** | **CP_CBCL_** | **CP_YSR_** | **ADHD_CBCL_^a^** | **ADHD_YSR_** |
| Participants’ mean earnings | 1 | -0.87** | -0.93** | 0.23 | 0.15 | 0.11 | 0.12 | -0.04 | -0.18 |
| Mean final Red Cross donation |  | 1 | 0.97** | -0.28 | -0.24 | -0.07 | -0.11 | 0.09 | 0.05 |
| Costly Helping |  |  | 1 | -0.28 | -0.22 | -0.09 | -0.09 | 0.09 | 0.15 |
| ICU_Total_ |  |  |  | 1 | 0.10 | -0.08 | 0.07 | -0.03 | 0.11 |
| CDSx |  |  |  |  | 1 | -0.11 | 0.28 | -0.25 | 0.17 |
| CP_CBCL_ |  |  |  |  |  | 1 | 0.08 | 0.63** | 0.003 |
| CP_YSR_ |  |  |  |  |  |  | 1 | 0.12 | 0.56** |
| ADHD_CBCL_ |  |  |  |  |  |  |  | 1 | 0.18 |
| **WITHIN CONTROLS (n=26)** | | | | | | | | | |
|  | **Participants’ mean earnings** | **Mean final Red Cross donation** | **Costly Helping** | **ICU_Total_** | **CDSx** | **CP_CBCL_** | **CP_YSR_** | **ADHD_CBCL_^a^** | **ADHD_YSR_** |
| Participants’ mean earnings | 1 | -0.88** | -0.88** | 0.27 | 0.20 | 0.35 | 0.18 | -0.09 | 0.26 |
| Mean final Red Cross donation |  | 1 | 0.98* | -0.30 | -0.28 | 0.51** | -0.09 | 0.05 | -0.24 |
| Costly Helping |  |  | 1 | -0.26 | -0.26 | -0.48* | -0.05 | 0.08 | -0.19 |
| ICU_Total_ |  |  |  | 1 | 0.25 | 0.37 | 0.26 | 0.07 | 0.22 |
| CDSx |  |  |  |  | 1 | 0.17 | 0.38 | -0.08 | 0.08 |
| CP_CBCL_ |  |  |  |  |  | 1 | 0.25 | 0.29 | 0.15 |
| CP_YSR_ |  |  |  |  |  |  | 1 | 0.08 | 0.37 |
| ADHD_CBCL_ |  |  |  |  |  |  |  | 1 | 0.26 |

*p<0.05; ** p<0.01. ^a^ = Not all parents completed the CBCL (n=66 across groups, 41 patients, 25 controls)

**Abbreviations:** ADHD_CBCL_=Diagnostic and Statistical Manual-oriented attention-deficit hyperactivity problems raw score from the Child Behavior Checklist; ADHD_YSR_=Diagnostic and Statistical Manual-oriented attention-deficit hyperactivity problems raw score from the Youth Self Report; CDSx = Whole life conduct disorder symptom count; Costly Helping = the number of Active Trials not taken by participants (range 0-72); CP_CBCL_ = Diagnostic and Statistical Manual-oriented conduct problems scale raw score from the Child Behavior Checklist; CP_YSR_ = Diagnostic and Statistical Manual-oriented conduct problems scale raw score from the Youth Self Report; ICU_Total_ = total score from the Inventory of Callous and Unemotional Traits.
